# Sarcoidosis: Immunopathogenesis and Immunological Markers

**DOI:** 10.1155/2013/928601

**Published:** 2013-07-25

**Authors:** Wei Sheng Joshua Loke, Cristan Herbert, Paul S. Thomas

**Affiliations:** ^1^Inflammation and Infection Research Centre, Faculty of Medicine, University of New South Wales, Sydney, NSW 2052, Australia; ^2^Department of Respiratory Medicine, Prince of Wales Hospital, Randwick, NSW 2031, Australia

## Abstract

Sarcoidosis is a multisystem granulomatous disorder invariably affecting the lungs. It is a disease with noteworthy variations in clinical manifestation and disease outcome and has been described as an “immune paradox” with peripheral anergy despite exaggerated inflammation at disease sites. Despite extensive research, sarcoidosis remains a disease with undetermined aetiology. Current evidence supports the notion that the immune response in sarcoidosis is driven by a putative antigen in a genetically susceptible individual. Unfortunately, there currently exists no reliable biomarker to delineate the disease severity and prognosis. As such, the diagnosis of sarcoidosis remains a vexing clinical challenge. In this review, we outline the immunological features of sarcoidosis, discuss the evidence for and against various candidate etiological agents (infective and noninfective), describe the exhaled breath condensate, a novel method of identifying immunological biomarkers, and suggest other possible immunological biomarkers to better characterise the immunopathogenesis of sarcoidosis.

## 1. Introduction

Sarcoidosis is a multisystem, inflammatory disorder of obscure aetiology. Its defining histopathology is the existence of noncaseating epithelioid granulomas with accompanying mononuclear cell infiltration and microarchitecture destruction [1, 2].

Although sarcoidosis involves the lungs in >90% of cases, it also affects the heart, skin, eye, and central nervous system [3]. This accounts for its heterogeneous clinical manifestation which ranges from having no symptoms to severe consequences, namely respiratory insufficiency, cardiac death, neurological disease, and blindness [4]. 

Sarcoidosis has been reported in all ethnic and racial groups with the majority of studies recording a peak incidence of 20–39 years of age for both males and females and a bimodal distribution whereby women have another peak incidence at 65–69 [4]. 

Disease remission occurs in as many as two-thirds of patients, usually in the first 3 years after diagnosis. Other patients have chronic unremitting sarcoidosis which may subsequently lead to lung fibrosis [1]. The erratic clinical course has impelled research into biomarkers that could delineate disease severity and outcome [5]. To date, there exist no reliable and practical biomarkers for sarcoidosis [6]. Moreover, despite earnest research efforts, the immunopathogenesis and aetiology underpinning sarcoidosis remains elusive [3]. 

This review outlines the current understanding of sarcoidosis, with reference to *ex vivo* lymphocyte stimulation in the peripheral blood and bronchoalveolar lavage fluid (BALF) of sarcoidosis patients, describes the exhaled breath condensate, an innovative method of identifying immunological markers, and proposes novel immunological markers to better characterise the immunopathogenesis of sarcoidosis.

## 2. Immunopathogenesis of Sarcoidosis

### 2.1. Key Features of the Pathological Process

#### 2.1.1. Immune Paradox

Sarcoidosis can be described as an “immune paradox.” Peripheral anergy is observed despite exaggerated inflammation at disease sites [7]. This is demonstrated by a reduced delayed-type hypersensitivity to tuberculin and common antigens [8]. It has been postulated that underpinning this paradoxical situation is a disequilibrium between effector and regulatory lymphocytes (T_reg_ cells), notably CD4^+^CD25^bright^FoxP3^+^ cells [7]. These cells accumulate in the periphery of the granuloma and peripheral blood of patients with active disease and exert anti-proliferative effects on naïve T cells. They only weakly suppress TNF*α* production [9], therefore allowing granuloma formation. Others argue that the intense immune response at disease sites results in activated T cells gathering at these disease sites and consequent peripheral blood lymphopenia [10]. Still others have suggested that with disease chronicity, immunosuppressive CD8^+^ T cells become more abundant peripherally, resulting in an anergic response [11].

#### 2.1.2. Granuloma Formation

The noncaseating epithelioid granuloma is the histologic hallmark of sarcoidosis. Its centre is hypothesized to contain a poorly degraded antigen, surrounded by macrophages that will differentiate to epithelioid cells which subsequently fuse to form multinucleated giant cells. CD4^+^ T helper cells are interspersed in the granuloma while CD8^+^ T cells, regulatory T cells, fibroblasts, and B cells surround the periphery [4, 12]. Birefringent crystals, Hamazaki-Wesenberg, Schaumann bodies, and asteroid bodies may also be present but are nonspecific [13].

### 2.2. Immune Reactions in Sarcoidosis

#### 2.2.1. Antigen Presentation

**Figure 1:**
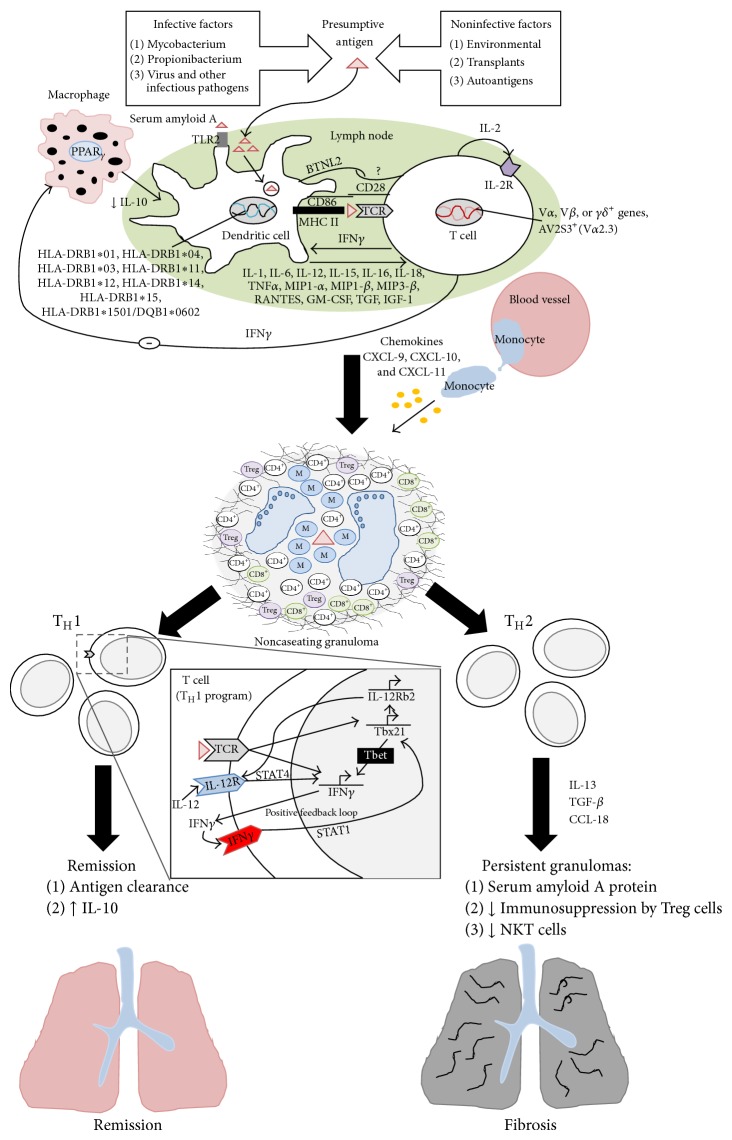
The immunopathogenesis of sarcoidosis (a proposed model). The presumptive sarcoid antigen is engulfed by circulating dendritic cells. Serum amyloid A proteins can also interact with Toll-like receptor 2 and be presented to T cells via major histocompatibility complex Class II to specific T cell receptors (TCRs) along with processed antigen peptides. Ligation of costimulatory molecules CD28, CD86, and BTNL2 optimises the activation of T cells. Thereafter, a myriad of inflammatory mediators is released. Activated T cells are highly T_H_1 polarised. They release IL-2 which causes clonal proliferation of T cells. Furthermore, upon TCR activation, T-bet production increases. T-bet upregulates and perpetuates the production of IFN*γ* which facilitates granuloma formation. Antigen clearance and increased IL-10 levels facilitate disease remission. Disease chronicity results in a predominance of T_H_2 cytokines which leads to lung remodelling by fibrosis (adapted from: [2, 5, 12, 14–19]).

The interplay of antigen-presenting dendritic cells (DCs) and naïve CD4^+^ T-cells is necessary for granuloma formation [18]. DCs phagocytose the inciting sarcoid antigen. They journey to lymph nodes where they mature and prime the adaptive immune system by displaying the antigen peptide on the surface major histocompatibility complex (MHC) class II peptide groove. A specific T cell receptor (TCR) fixes its variable region to the antigen-MHC complex and is activated [18]. To optimise this activation, CD28, a costimulatory signalling molecule on T cells, interacts with CD86 on DCs [15]. The DCs also produce a battery of mediators which facilitates the sarcoid immune reaction (Figure 1) [20].

#### 2.2.2. T-Helper 1 (T_H_1) Immune Response

The CD4^+^ T cells that trigger the granuloma formation are strongly T_H_1 polarised. Upon TCR activation, the expression of IFN*γ* and Tbx21 genes in CD4^+^ T cells becomes more pronounced. Interleukin-12 (IL-12) secreted by DCs is a T_H_1 polarising cytokine. With the aid of STAT4, IL-12 facilitates IFN*γ* expression. IFN*γ* binds to IFN receptors and stimulates STAT1 which promotes Tbx21 gene expression of T-bet. T-bet enhances IFN*γ* gene transcription competence and ultimately increases the production of IFN*γ* (Figure 1). T-bet also up regulates IL-12 *β* receptor (IL-12*β*R) expression and antagonises Gata3, a transcription factor that regulates T_H_2 differentiation. This amplifies the responsiveness of CD4^+^ T-cells to IL-12 and inhibits IL-4 and IL-13 (cytokines that facilitate the fibroproliferative response) production [16]. IL-18 upregulates IL-12*β*R and IFN*γ* expression while IL-12 increases IL-18 receptor expression on CD4^+^ T cells. Therefore, IL-12 and IL-18 act synergistically to promote the formation of sarcoid granulomas [20–23].

IFN*γ* is highly expressed in the BALF of sarcoidosis patients. IFN*γ* inhibits the expression of macrophage peroxisome proliferator-activated receptor *γ* (PPAR*γ*), a negative regulator of inflammation. Under normal physiological conditions, macrophages constitutively express PPAR*γ*. PPAR*γ* promotes macrophage IL-10 production which inhibits the release of TNF*α*, IL-12 and matrix metalloproteinase (MMP) from DCs. In sarcoidosis, IFN*γ* production inhibits the expression of the immunosuppressive cytokine, IL-10 (Figure 1). This leads to an increase in the production of TNF*α*, IL-12, and MMP and induces chemokines CXCL-9, CXCL-10, and CXCL-11 production which, through the ligation of a T-cell receptor, CXCR3, induce T-cell chemotaxis. MMPs cause lung damage and fibrosis and the chemokines attract more Tcells and myeloid cells into the inflammatory milieu. Moreover, increased TNF*α* and decreased IL-10 expression liberate DCs from the inhibition by macrophages, initiating a self-amplifying inflammatory loop [19].

TNF*α* produced by the DCs also encourages CD4^+^ T-cell proliferation and survival, directly through the induction of T-cell IL-2R [24, 25] and indirectly by causing DC to mature into antigen presenting cells [26]. IL-15 is capable of promoting CD4^+^ T-cell survival by binding to IL-2R. These IL-15 responses are upregulated in the presence of TNF*α* [27].

Finally, CD4^+^ T-cell activation also increases IL-2 production. IL-2 is a local survival, differentiation, and growth factor of T cells. Autocrine IL-2 production results in the clonal proliferation of CD4^+^ T cells (Figure 1) [2, 28].

### 2.3. Persistent Granulomatous Inflammation

Persistent granulomatous inflammation can be attributed to the inability of the immune regulatory mechanisms to limit the duration of the inflammatory process [12]. 

#### 2.3.1. Serum Amyloid A Protein

Serum amyloid A (SAA) proteins are extensively deposited in sarcoid granulomas. SAA triggers cytokine release by interacting with Toll-like receptor 2. This results in the amplification of T_H_1 responses to local pathogenic antigens. The inflammatory response is potentiated as SAA proteins readily accumulate and release more soluble SAA peptides into the surrounding tissue [29, 30].

#### 2.3.2. T Regulatory (T_reg_) Cells

T_reg_ cells are vital for the suppression of cell-mediated immune responses. However, the T_reg_ cells in the sarcoid granulomas (as opposed to peripheral T_reg_ cells) have undergone extensive amplification and are therefore impaired in their ability to repress immune responses. Moreover, they secrete proinflammatory cytokines (e.g., IL-4) which encourages granuloma formation via mast cell activation and fibroblast amplification [31, 32].

#### 2.3.3. CD1d-Restricted Natural Killer T (NKT) Cells

NKT cells have been known to moderate CD4-mediated immune responses. NKT cell numbers have been noted to be markedly reduced in sarcoid blood and BALF except in patients exhibiting Löfgren's syndrome (acute sarcoidosis characterised by uveitis, arthritis, erythema nodosum, bilateral hilar lymphadenopathy, and fever) [33]. Since Löfgren's syndrome is often associated with disease remission, reduction in the number of NKT cells can account for the persistence of sarcoidosis [34].

### 2.4. Remission and Progression to Fibrosis

Disease remission occurs with the suppression of macrophage and T-helper cell activity by IL-10 or when the presumptive antigen has been completely cleared (Figure 1) [4]. 

Persistent granulomatous inflammation can lead to fibrosis. The immunological mechanisms leading to a fibrotic outcome remain undetermined. Nonetheless, various cytokines which are able to support a fibrotic response have been found at disease sites in patients with sarcoidosis (e.g., transforming growth factor-*β* (TGF-*β*), MMP, and insulin growth factor-1 (IGF-1)) [35, 36]. 

It has been proposed that a switch from T_H_1 to T_H_2 cytokine predominance may occur in chronic sarcoidosis in response to persistent inflammation. T_H_2 cytokines such as IL-13 increase TGF-*β* production (Figure 1). TGF-*β* recruits, activates, and transforms fibroblasts into myofibroblasts which have been strongly implicated in the development of fibrosis [17, 37]. Moreover, the T_H_2 chemokine, CCL2, enhances fibroblast survival, augmenting the effects of TGF-*β* [38]. Additionally, the macrophages of patients with pulmonary fibrosis, under the influence of the T_H_2 cytokine milieu, express CCL18 chemokines which facilitates lung remodelling via fibrosis [39, 40]. 

### 2.5. Role of Other T Lymphocytes (T_H_17 and NKT Cells)

Although the majority of studies have used the T_H_1/T_H_2 model to explain the immunopathogenesis of sarcoidosis, by focusing solely on this model, there is a propensity to oversimplify the immunological process and divert research efforts away from other mechanisms.

T_H_17 is a novel CD4^+^ effector T-cell population. High levels of IL-17^+^/CD4^+^ T lymphocytes have been found in the BALF and granulomas of sarcoidosis patients, particularly in patients with active disease. They infiltrate the lungs after being recruited from the blood by the chemokine CCL20 [41]. Recently, Richmond and colleagues [42] verified the specificity of T_H_17 cells for mycobacterial antigens, a commonly implicated antigen for sarcoidosis. These findings suggest a possible role of T_H_17 in sarcoidosis disease progression.

NKT cells produce T_H_1 and T_H_2 cytokines (IFN*γ* and IL-4, resp.). NKT cells are mostly CD4^+^ and express an invariable TCR [33]. Moreover, blood NKT cells from sarcoidosis patients, when stimulated with a glycolipid stimulator, showed diminished levels of IFN*γ*, therefore suggesting that NKT cells exert regulatory activity which prevents disease progression [43].

## 3. Putative Aetiology of Sarcoidosis

The aetiology of sarcoidosis remains unclear. A myriad of observations have supported the notion that sarcoidosis can be caused by environmental and infectious agents. Moreover, based on chronic beryllium disease, an analogous granulomatous lung disease, it has been speculated that one or more antigenic stimuli may be involved in the pathogenesis of sarcoidosis. Therefore, it is highly likely that the development of a sarcoidosis reaction to an antigen depends on a combination of genetic polymorphisms, the host's immune status, and exposure to environmental agents [44]. 

### 3.1. Genetic Polymorphisms and Host Factors

#### 3.1.1. Findings on Genome-Wide Association

Both family and genetic host studies have recognised genes that are responsible for this genetic susceptibility. Twin studies prove that monozygotic twins are more concordant for sarcoidosis than dizygotic twins. Moreover, familial aggregation of sarcoidosis can be seen worldwide. The multicentre study entitled *A Case Control Etiologic Study of Sarcoidosis* (ACCESS) demonstrated that sarcoidosis patients were 5 times more probable than controls to report a parent or sibling with sarcoidosis [45].

Genetic linkage studies on German families revealed a strong linkage to chromosome 6p. This led to the discovery of butyrophilin-like 2 (BTNL2), a costimulatory molecule within the MHC locus. Single nucleotide polymorphisms (rs2076530 G → A) in BTNL2 may affect T-cell regulation and activation [45].

The genome-wide association study conducted by Hofmann and colleagues [46] revealed an association for the annexin A11 gene located on chromosome 10q22.3. The annexin A11 gene regulates calcium signalling, vesicle trafficking, cell division, and apoptosis. Therefore, its dysfunction or deletion may implicate apoptotic pathways in sarcoidosis [46].

#### 3.1.2. Human Leukocyte Antigen (HLA) Genes

HLA class II are cell surface proteins that prime the adaptive immune system to antigens. Sarcoidosis is associated with the DR subtypes of class II antigens. HLA-DRB1∗01 and HLA-DRB1∗04, are negatively associated with sarcoidosis, whereas HLA-DRB1∗03, HLA-DRB1∗11, HLA-DRB1∗12, HLA-DRB1∗14 and HLA-DRB1∗15 have been shown to increase the risk of sarcoidosis. HLA-DRB1∗03 is associated with Löfgren's syndrome (~80% of patients with Löfgren's syndrome experience disease remission). Finally, the HLA-DRB1∗1501/DQB1∗0602 haplotype was associated with severe and chronic pulmonary sarcoidosis [6, 47].

#### 3.1.3. Non-HLA Genes

TNF*α* is an essential mediator for granuloma formation. Variants of the TNF gene confer a 1.5-fold increased risk of having sarcoidosis [48]. Apart from TNF, studies investigating other candidate genes (polymorphisms in the complement receptor 1 gene, NOD, and CCR2 genes) were inconclusive and had poor reproducibility between populations [49–52].

In some populations, variations in the gene that encodes for RAGE (a transmembrane receptor) have been associated with an increased risk of sarcoidosis. However, the close proximity of this gene to the MHC region makes it difficult for one to ascertain if this association is due to linkage with neighbouring HLA genes [53].

There were no associations between polymorphisms in genes for vitamin D receptor or serum angiotensin-converting enzyme [45, 54].

#### 3.1.4. T Cell Receptor (TCR) Genes

The T-cells at sites of inflammation in sarcoidosis exhibit a restricted repertoire of TCR *γδ* or *αβ* genes. The expression of specific V*α*, V*β*, or *γδ*+ TCR genes in blood, lung, and at sites of Kveim-Siltzbach skin reactions implies that sarcoidosis is an antigen-driven disorder. There is a subpopulation of T cells (AV2S3^+^ (V*α*2.3) CD4^+^ T cells) from BALF of HLA-DR∗0301 sarcoidosis patients which is unique to sarcoidosis. Moreover, it has been shown that the amount of AV2S3^+^ BALF T cells at the onset of sarcoidosis correlates positively with prognosis, suggesting that AV2S3^+^ T cells may offer some protective function against sarcoidosis [6].

### 3.2. Extrinsic Factors

Numerous pathogens have been implicated and investigated in the etiology of sarcoidosis. Moreover, spatial crowding of unrelated sarcoidosis cases suggests that sarcoidosis can also be a result of exposure to environmental agents [1, 3]. Nonetheless the evidence supporting specific infectious and environmental factors varies significantly (Table 1) [3]. 

## 4. *Ex Vivo* Stimulation of BALF and Peripheral Blood Lymphocytes

Preliminary *ex vivo* studies that employed flow cytometry to investigate peripheral blood lymphocytes in sarcoidosis patients demonstrated a greater activation of nonstimulated CD4^+^ and CD8^+^ BALF T cells compared to peripheral blood lymphocytes. This showed that the sarcoid immune response is largely compartmentalised to disease sites [55]. 

The T_H_1/T_H_2 model is under scrutiny as it oversimplifies the immunopathogenesis of sarcoidosis [56]. For instance, some studies report that after lymphocyte stimulation, the proportion of CD4^+^ T cells expressing IL-4 and IFN*γ* obtained from the peripheral blood of sarcoidosis patients did not differ significantly from that of healthy controls [57, 58]. Other studies showed higher T_H_1 and T_H_2 levels of cytokine positive CD4^+^ T cells compared to healthy controls [59, 60], emphasising the systemic nature of the disease. The following segment clarifies this debate.

Under unstimulated conditions, the difference in the percentages of IL-4 and IFN*γ* secreting CD4^+^ lymphocytes in BALF and peripheral blood of sarcoidosis patients is insignificant [57, 58]. After BALF CD4^+^ lymphocytes were stimulated with ionomycin and phorbol 12-myristate acetate, there was an appreciable increase in secreted IFN*γ* but a decrease in IL-4 expression in sarcoidosis patients compared to controls [87]. Moreover, increased cytokine profiles have been verified by increased BALF IFN*γ* ^+^/IL-4^+^ CD4^+^ T cell ratios in sarcoidosis patients. Lower ratios were demonstrated in scleroderma and in patients with idiopathic pulmonary fibrosis. It has also been shown that upon stimulation, compared with controls, there are increased numbers of CD4^+^IFN*γ* ^+^ cells in both BALF and induced sputum of patients with sarcoidosis [88, 89].

After stimulation, more T cells express T_H_1 than T_H_2 cytokines in both the BALF and peripheral blood of sarcoidosis patients and more CD4^+^ T cells in BALF express T_H_1 receptors (CXCR3, CCR5, IL-12R and IL-18R) than CD4^+^ T cells in the peripheral blood. [90]. Although CD4^+^ T cells largely express T_H_1 cytokines, interestingly, following stimulation, only 80% and 40% of CD4^+^ IL-4^+^ cells concurrently produce IFN*γ* and IL-2 respectively, thus demonstrating that activated BALF lymphocytes of sarcoidosis patients are capable of a complex, concurrent production of T_H_1 and T_H_2 cytokines [91]. 

To further explore this dichotomy of blood T_H_1/T_H_2 equilibrium, Nureki and colleagues [59] showed that under unstimulated conditions, T_H_1 and T_H_2 chemokines (interferon-inducible protein-10 (IP-10) and thymus and activation-regulated chemokine (TARC)) were both increased in the serum of sarcoidosis patients. This was in agreement with previous findings that demonstrated elevated BALF and peripheral blood IL-13 (a T_H_2 cytokine) mRNA levels [92]. Therefore, these findings reflect the systemic nature of sarcoidosis. Nonetheless, it has been suggested that T_H_2 cell preponderance occurs in the peripheral blood of sarcoidosis patients and that this, together with the generalised intensification of T_H_1 activity, gives the appearance of an increase in both T_H_1 and T_H_2 circulating cytokine expression in sarcoidosis patients compared to healthy controls [59].

T_H_17 cells have also been implicated in the induction of granuloma formation [93]. Flow cytometry data indicate that after stimulation, there is an increase in T_H_17 related cytokine levels in both BALF and peripheral blood [41]. Another study also indicated that, after stimulation, there are lower levels of IL-17A gene expression in CD4^+^ T cells in patients with Löfgren's syndrome compared to healthy controls [94]. These data, together with data showing heightened T_H_1 cytokine expression at disease sites, indicate that T_H_17 cells have a systemic role in patients with non-Löfgren's disease and is involved in sarcoidosis progression [14]. Therefore, further studies investigating the cytokine profiles in blood lymphocytes of patients versus healthy controls are required to assess the T_H_1/T_H_2 balance, the regulatory mechanisms in the peripheral blood of sarcoidosis patients, and the functional significance of T_H_17 cells.

## 5. Exhaled Breath Condensate: Detection of Immunological Markers

The diagnosis of sarcoidosis is never secure. Clinico-radiological findings alone are often insufficient to confirm the diagnosis of sarcoidosis. It needs to be supported by histological evidence showing noncaseating granulomas. This warrants a tissue biopsy which is invasive [12]. This makes diagnosing sarcoidosis a vexing clinical challenge, motivating researchers to look for other novel methods of diagnosing the disease.

**Figure 2:**
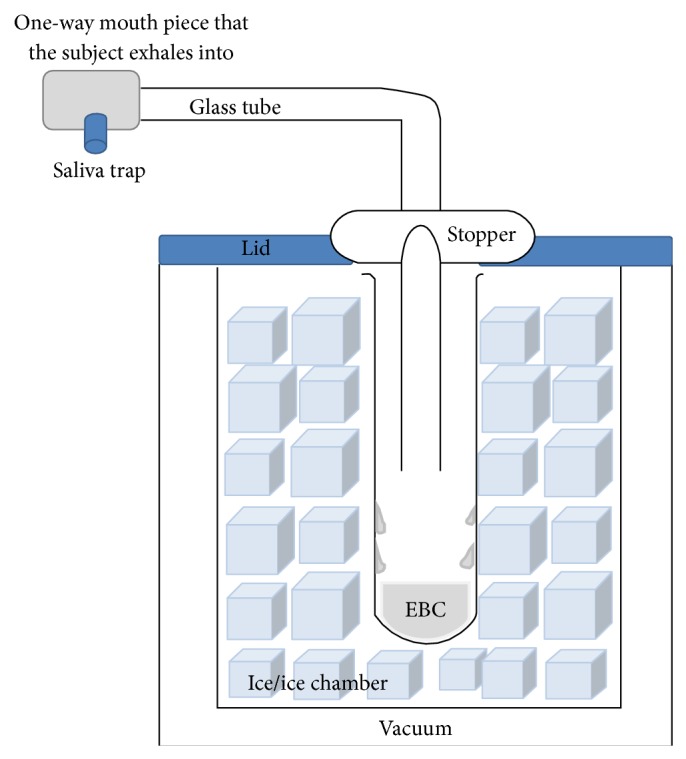
Schematic diagram of the EBC collecting apparatus. The subject blows into the mouth piece which is a one-way valve. The exhaled breath is channelled into a refrigerated collecting container.

Exhaled breath condensate (EBC) has been subjected to intensive research as it provides a noninvasive alternative for sampling the airway and alveolar space a promising source of biomarkers for a variety of lung conditions [95–97]. During exhalation, water evaporation droplets and volatile molecules (e.g., nitric oxide, carbon monoxide and hydrocarbons) diffuse as gases from the alveoli and bronchi to the mouth. They are joined by nonvolatile molecules (e.g., leukotrienes, prostanoids, urea, and cytokines) from the airway lining fluid and condense via a refrigeration device to give EBC (Figure 2) [95, 98]. 

A number of immunological biomarkers have been recognised in EBC. However, there exists no sufficiently sensitive and specific marker for diagnosing and predicting the prognosis of sarcoidosis [99]. 

TNF*α*, PAI-1, and IGF-1 levels in EBC were closely positively correlated with BALF samples from sarcoidosis patients. Conversely, IL-6 levels were negatively correlated with that which is in BALF. The propensity of IL-6 to form complex molecular forms of higher molecular weight could account for this discrepancy [100]. Another study detected TGF-*β*_1_, PAI-1, TNF*α*, IL-8 and vascular endothelial growth factor in sarcoid EBC. However, the small sample size and the failure to make comparisons with healthy controls limited the usefulness of this study [101].

Exhaled eicosanoids (e.g., 8-isoprostane (8-IP)) and cysteinyl leukotrienes were also found to be elevated in the BALF and EBC of sarcoidosis patients [102]. In a later study, high initial levels of 8-IP were shown to correlate with disease persistence; therefore, it could serve as a prognostic marker [103].

Cellular and molecular biomarkers previously discovered in BALF and serum of sarcoidosis patients could also serve as biomarkers. These include eosinophils, neutrophils, serum angiotensin converting enzyme (ACE), neopterin, chitotriosidase, TGF-*β*, and the chemokine ligand (CCL18) [28, 40, 57, 104–107]. Other more novel markers include lysozyme, Kerbs von Lungren 6 antigen, and soluble IL-2 receptor [108, 109]. Serum levels of these biomarkers were said to reflect increased parenchymal infiltration and lymphocytic alveolitis in sarcoidosis and can thus serve as potential EBC biomarkers. Nonetheless, only a few have been shown to be sufficiently sensitive and specific [110]. 

Amongst the above-mentioned biomarkers, ACE is the most contentious as it has been shown to have poor sensitivity and specificity [111] and its activity is subject to the effects of gene polymorphisms. Nevertheless, it is elevated and measurable in the BALF of sarcoidosis patients and could therefore serve as a sarcoid biomarker [112].

Given the multifactorial nature of sarcoidosis, no ideal markers for detecting and monitoring the clinical course of sarcoidosis exist. It is very likely that a combination of markers will be required. 

Although EBC has advantages over BAL (it is noninvasive, requires little instrumentation, does not introduce foreign substances into the lung or cause inflammatory changes, and can be repeatedly performed in sick patients) [113], the lack of reliable markers and the inability of EBC to sample specific compartments of the lungs undermine these benefits. To date, BALF remains the most relevant biological material.

## 6. Interferon Modulators: Novel Immunological Markers

IFN*γ* plays a pivotal role in the immunopathogenesis of sarcoidosis. MicroRNA-29 (miR-29) and T-bet have been shown to modulate its production [114, 115]. 

MicroRNAs are noncoding RNA that can inhibit the production of mRNA. The miR-29 family is made up of four members. Amongst these four, miR-29a and miR-29b were found to be downregulated in IFN*γ*-secreting T cells. This reduction skews the immunological response towards a T_H_1 lineage by initiating a positive feedback loop which enhances IFN*γ* production. This also suggests that the up-regulation of miR-29a and miR-29b can mitigate IFN*γ* expression [115, 116]. Abnormal levels of microRNA have been associated with the pathogenesis of cancers and fibrotic and obstructive lung diseases [117–119]. It has also been implicated in the fibrotic progression of sarcoidosis [120]. 

As previously mentioned (see immune reactions in sarcoidosis), T-bet is a transcription factor necessary for IFN*γ* production. It binds to a number of enhancers and to the promoter region of the IFN*γ* gene to promote IFN*γ* transcription [121]. T-bet expression has been shown to correlate with IFN*γ* expression [16, 122, 123] in patients with multiple sclerosis [114], coeliac disease [124], Crohn's disease [125], and Behçet's disease [126, 127]. Moreover, T-bet mRNA has been shown to be elevated in the BALF lymphocytes of patients with pulmonary sarcoidosis [128]. However, due to posttranscriptional regulation and disparities in protein and mRNA turnover rates, mRNA levels are poor proxies for protein levels [129, 130]. Unfortunately, the literature is currently deficient of studies that measure T-bet protein levels in sarcoidosis patients and studies that juxtapose miR-29 and T-bet protein levels at sarcoidosis disease sites and in the peripheral blood. Research on these fronts can offer novel insights into the “immune paradox” associated with sarcoidosis and can pave the path for novel therapeutic strategies for the disease.

## 7. Conclusion

Despite nearly 140 years of extensive research, the aetiology and pathogenesis of sarcoidosis and the mechanisms that regulate the immune reactions in the peripheral blood remain undetermined. Moreover, given its variable clinical manifestation and the lack of a reliable diagnostic test with uniformed reference values and measurements, diagnosing sarcoidosis remains a clinical conundrum for many physicians. Given that the majority of sarcoidosis patients have pulmonary involvement, EBC could be used as a non-invasive method to diagnose sarcoidosis. Besides being a potential immunological biomarker of sarcoidosis, interferon modulator levels in EBC and the peripheral blood can be compared to elucidate the regulatory mechanisms in the peripheral blood. Results from such studies may also explain the pathology underpinning the peripheral anergy seen in sarcoidosis.

## Figures and Tables

**Table 1:** List of infectious and noninfectious agents and the evidence for and against them.

Nature	Causative agent	Evidence for	Evidence against
Infective	Mycobacterium	(i) Immunohistochemical studies showed possible remnants of cell wall deficient mycobacteria [61]. (ii) A mycobacterial cell wall component, tuberculostearic acid, was found in sarcoid specimens [62]. (iii) Techniques such as enzyme-linked immunospot assay (ELISpot) and polymerase chain reactions (PCR) have shown increasing evidence for mycobacteria in the mediastinal lymph nodes and peripheral lung tissues of sarcoidosis patients [63]. (iv) Mycobacterium tuberculosis DNA: mycobacterium tuberculosis catalase-peroxidase protein (mKatG) and circulating IgG for mKatG have been identified in sarcoidosis patients [64]. Additionally, compared to healthy controls, sarcoidosis patients have amplified T-cell responses in the peripheral blood and lungs to mKatG and mycobacteria antigens [65, 66]. (v) Mycobacterial heat-shock proteins (Mtb-hsp)70, 65, and 16 were found in the lymph nodes and sera of sarcoidosis patients [67]. (vi) Dubaniewicz and colleagues (2013) suggested that, in genetically different individuals, Mtb-hsp 16 can induce an autoimmune response in sarcoidosis.	(i) Acid-fast stains and cultures of sarcoid specimens do not routinely demonstrate the presence of *mycobacterium* species. (ii) The mere presence of the mycobacterial antigens in sarcoid specimens is not proof of a causal relationship. (iii) Mycobacterial nucleic acid and antigens are not detected in many sarcoid specimens; therefore, mycobacteria may not be the sole cause of sarcoidosis [3].
	Propionibacterium	(i) It has been shown to be able to induce a granulomatous reaction [68]. (ii) *P. acnes* has been found in up to 78% of sarcoidosis sample cultures [69]. (iii) An antibody response to *P. acnes* proteins has been observed in 40% of BALF samples (compared to 5% in healthy controls) [70].	(i) Cultures from healthy controls also yield this commensal organism [71].
	Viruses and other infectious pathogens	(i) Serum antibodies to herpes-like viruses (human herpes virus-8, herpes simplex virus, and Epstein-Barr virus) were elevated in patients with sarcoidosis [72].	(i) Significant proportions of the general population have also been previously exposed to herpes-like viruses. (ii) A nonspecific polyclonal hypergammaglobulinemia, common in sarcoidosis, may explain the increased antibody titre for these viruses [73]. (iii) Viruses do not cause the epithelioid granulomas of sarcoidosis [74]. (iv) The mechanism for granuloma formation by molecular mimicry after viral exposure remains undetermined [74]. (v) Granulomatous reactions resulting from spirochetes, fungi, *Tropheryma whipplei,* and Borrelia species infection can be difficult to discriminate from sarcoidosis [75].
			(vi) The notion that cell-wall deficient organisms like mycobacteria, rickettsia, and chlamydia species cause sarcoidosis is founded on limited data. There is a shortage of conformation from well-controlled epidemiological and laboratory studies [75].
	Transplants	(i) Immune dysregulation following allogeneic hematopoietic cell transplantation has been shown to promote sarcoidosis in patients with susceptible HLA subtypes [76]. (ii) Individuals have developed granulomatous inflammation post lung and heart transplant from patients with sarcoidosis [77–79]. (iii) An increased incidence of sarcoidosis in closed populations may suggest an infection-related disease.	

Noninfectious	Environmental	(i) Wood stoves and fireplaces have been associated with an increased risk of sarcoidosis [80]. (ii) Fire rescue workers, military personnel, and healthcare workers who were exposed to the dust from the destruction of the World Trade Centre in New York were found to have a higher risk of developing sarcoidosis [81, 82]. (iii) Findings from the ACCESS study showed the modest positive odds ratios (~1.5) that workplace exposure to organic solvents, dusts, pesticides, insecticides, and musty odours can increase one's risk of sarcoidosis. Reduced risk was associated with exposures to animal dander and other allergic (T_H_2) responses [83]. (iv) Nanoparticles of common minerals and metals can elicit a dysregulated immune response [84].	(i) The ACCESS study failed to identify risk factors that accrue a greater than two-fold risk (odds ratio). Moreover, it had inadequate power to ascertain the sarcoidosis risk among fire rescue workers, military personnel, and healthcare workers. Lastly, it failed to prove an association between previously hypothesised exposures (e.g., wood dust, metals, and silica) and sarcoidosis [1, 3].
	Autoantigens	(i) Sarcoidosis patients express low titre levels of autoantibodies. (ii) The BALF of HLA-DRB1∗0301-positive sarcoidosis patients with Löfgren's syndrome had antigenic peptides that were bound to HLA-DR molecules of lung cells that have the TCR AV2S3^+^ gene segment. These antigenic peptides include vimentin, ATP synthase, and lysyl tRNA synthetase, thought to be autoantigens in various conditions [85]. (iii) IFN*γ* enzyme-linked immunospot assays revealed a strong T-cell response to the cytoskeletal peptides of vimentin from the peripheral blood of patients with HLA-DRB1∗0301. The same was observed for ATP synthase and lysyl tRNA synthetase from BALF. Thus, this suggests a possible autoimmune response in patients with HLA-DRB1∗0301, contributing to sarcoid granulomatous inflammation [86].	(i) The pathological significance of autoantibodies in sarcoidosis remains unclear. The disease-specific autoantibody profile has not been described. Therefore, it has been postulated that these autoantibodies are most likely the product of general B-cell stimulation in the progression of T-cell stimulation by antigens [1].
